# Association between methionine sulfoxide and risk of moyamoya disease

**DOI:** 10.3389/fnins.2023.1158111

**Published:** 2023-04-12

**Authors:** Junsheng Li, Peicong Ge, Qiheng He, Chenglong Liu, Chaofan Zeng, Chuming Tao, Yuanren Zhai, Jia Wang, Qian Zhang, Rong Wang, Yan Zhang, Dong Zhang, Jizong Zhao

**Affiliations:** ^1^Department of Neurosurgery, Beijing Tiantan Hospital, Capital Medical University, Beijing, China; ^2^China National Clinical Research Center for Neurological Diseases, Beijing, China; ^3^Center of Stroke, Beijing Institute for Brain Disorders, Beijing, China; ^4^Beijing Key Laboratory of Translational Medicine for Cerebrovascular Disease, Beijing, China; ^5^Beijing Translational Engineering Center for 3D Printer in Clinical Neuroscience, Beijing, China; ^6^Department of Neurosurgery, The Second Affiliated Hospital of Soochow University, Suzhou, China; ^7^Savaid Medical School, University of the Chinese Academy of Sciences, Beijing, China

**Keywords:** moyamoya disease, subtypes, methionine sulfoxide, risk factor, oxidative stress

## Abstract

**Objective:**

Methionine sulfoxide (MetO) has been identified as a risk factor for vascular diseases and was considered as an important indicator of oxidative stress. However, the effects of MetO and its association with moyamoya disease (MMD) remained unclear. Therefore, we performed this study to evaluate the association between serum MetO levels and the risk of MMD and its subtypes.

**Methods:**

We eventually included consecutive 353 MMD patients and 88 healthy controls (HCs) with complete data from September 2020 to December 2021 in our analyzes. Serum levels of MetO were quantified using liquid chromatography-mass spectrometry (LC–MS) analysis. We evaluated the role of MetO in MMD using logistic regression models and confirmed by receiver-operating characteristic (ROC) curves and area under curve (AUC) values.

**Results:**

We found that the levels of MetO were significantly higher in MMD and its subtypes than in HCs (*p* < 0.001 for all). After adjusting for traditional risk factors, serum MetO levels were significantly associated with the risk of MMD and its subtypes (p < 0.001 for all). We further divided the MetO levels into low and high groups, and the high MetO level was significantly associated with the risk of MMD and its subtypes (*p* < 0.05 for all). When MetO levels were assessed as quartiles, we found that the third (Q3) and fourth (Q4) MetO quartiles had a significantly increased risk of MMD compared with the lowest quartile (Q3, OR: 2.323, 95%CI: 1.088–4.959, *p* = 0.029; Q4, OR: 5.559, 95%CI: 2.088–14.805, *p* = 0.001).

**Conclusion:**

In this study, we found that a high level of serum MetO was associated with an increased risk of MMD and its subtypes. Our study raised a novel perspective on the pathogenesis of MMD and suggested potential therapeutic targets.

## Introduction

Moyamoya disease (MMD), as a chronic intracranial vasculopathy, was unknown for its etiology. MMD was characterized by the stenosis or occlusion at the terminal portion and main branches of internal carotid artery (ICA) associated with the formation of anomalous collateral circulation on angiography (Kuroda and Houkin, 2008). MMD was more prevalent in East Asian countries than in Europe and North America (Kim, 2016). Moreover, familial aggregation and *RNF213* variants suggested the genetic etiology in MMD (Miyatake et al., 2012; Kim et al., 2016; Zhang et al., 2017). Our previous study has revealed the correlation between several traditional modifiable risk factors and MMD (Ge et al., 2020). These findings indicated that genetic, inflammation, and environmental factors were involved in MMD. Currently, direct and indirect revascularizations have been the most routine treatment for MMD patients (Acker et al., 2018; Deng et al., 2018). However, the outcome after surgery varied widely, and the MMD process seemed irreversible. Therefore, it has been necessary to investigate novel risk factors in the pathogenesis and progression of MMD.

In recent years, with the further study of multi-omics technologies, researchers have found that the ultimate effect of gene or protein regulatory action was to cause changes in metabolites in body (Holmes et al., 2018; Yang et al., 2018; Zhang et al., 2022). Metabolomics studied endogenous small molecule compounds at the end of metabolic pathways, which could skip the complex regulatory processes of body and directly reflect the final physiological and pathological changes (Ke et al., 2018; Guo et al., 2020; Chumachenko et al., 2022). Through the analysis of differential metabolites, researchers could reveal pathogenic mechanisms, confirm diagnostic biomarkers, estimate disease risk, and monitor treatment processes (Wishart, 2019; Shin et al., 2020; Wang et al., 2020). Homocysteine (Hcy), one of the methionine (Met) related metabolites, has been identified associated with MMD and its prognosis (Ge et al., 2020; He et al., 2022). It indicated the important role of Met metabolism in MMD. Methionine sulfoxide (MetO), another Met-related metabolite, has been studied to be associated with neurodegenerative diseases, mental health diseases, obesity, cancers, and cardiovascular diseases (Lei et al., 2007; De Luca et al., 2010; Ma et al., 2011; Rose and Hoffmann, 2015; Jiang and Moskovitz, 2018). Furthermore, MetO could reflect the level of oxidative stress. However, the metabolomics studies on MMD were still limited, and the role of MetO in MMD has hardly been studied.

Therefore, this study aimed to demonstrate the correlation between MetO and the risk of MMD, contributing to the exploration of novel risk factors and the identification of potential therapeutic targets in MMD.

## Methods

### Study participants

**Figure 1:**
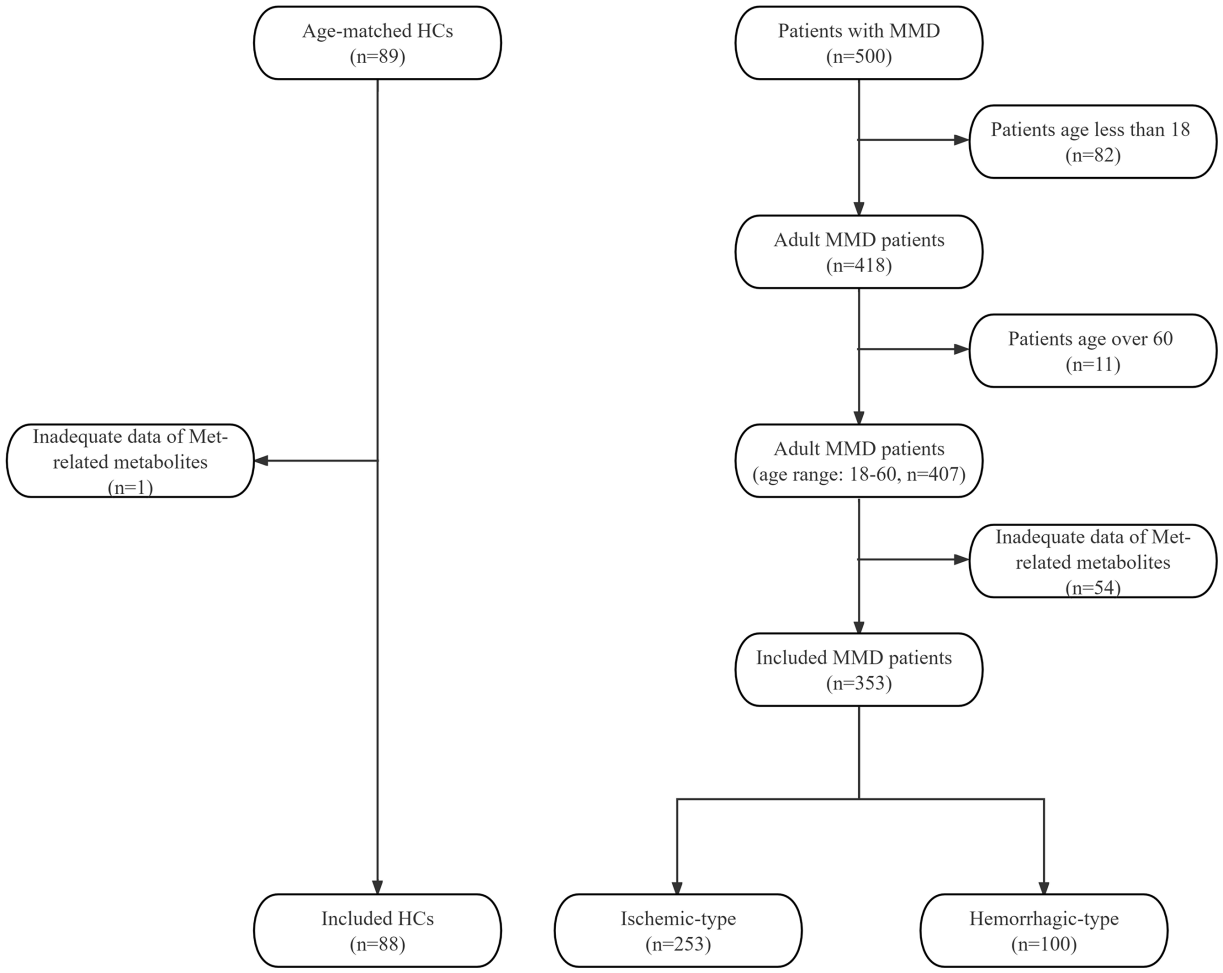
Flowchart of the study participant inclusion.

In this prospective study, we consecutively included MMD patients from September 2020 to December 2021 and collected the clinical data. All patients were diagnosed as MMD with digital subtraction angiography (DSA) according to the 2012 Japanese diagnostic criteria (Guidelines for diagnosis and treatment of moyamoya disease, 2012). Minors and patients without complete liquid chromatography-mass spectrometry (LC–MS) data of Met-related metabolites were excluded. We recruited 89 age-matched healthy individuals as the control group. All healthy controls (HCs) underwent routine examinations and were excluded cerebrovascular diseases. One HC with inadequate Met-related metabolite data was excluded. A total of 353 adult MMD patients (253 ischemic-type, 100 hemorrhagic-type) and 88 HCs were eventually included (Figure 1). All participants have provided written informed consent to participate in this research. This study has been approved by the Ethics Committee of Beijing Tiantan Hospital.

### Data collection

All baseline characteristics, including demographic data (age and gender), medical history (hypertension, diabetes, hyperlipidemia, smoking, drinking), clinical features (heart rate, systolic blood pressure [SBP], diastolic blood pressure [DBP], body mass index [BMI]) were collected by independent chart reviews. Fasting peripheral blood samples from all participants were used for routine and biochemical blood tests, as shown in Table 1. We also calculated peripheral inflammation indicators based on the laboratory examination data, which were also presented in Table 1 (Zeng et al., 2022). The peripheral blood samples were obtained on the day of admission, then centrifuged, separated, and aliquoted into separation tubes in 1 h. All blood samples were stored at −80°C until testing. LC–MS analysis has been used for MetO quantification, and the laboratory technicians were blinded to all patients’ data. We detected *RNF213* p.R4810K variant with primer designation of RNF213-4810F 5’-GCCCTCCATTTCTAGCACAC-3′ and RNF213-4810R 5’-AGCTGTGGCGAAAGCTTCTA-3′. Neurological status was assessed by the modified Rankin Scale (mRS) at admission and divided into two groups (0–2, 3–5). The Suzuki stage of MMD patients was defined as the more severe side.

**Table 1:** Baseline characteristics between HC and MMD groups.

Variables	Health Control (*n* = 88)	MMD (*n* = 353)	*p* value
Age, y, mean ± SD	39.75 ± 11.62	41.66 ± 10.27	0.160
Gender, female, *n* (%)	51 (58.0)	205 (58.1)	0.984
Medical history, *n* (%)			
Hypertension	0 (0)	129 (36.5)	<0.001*
Diabetes	0 (0)	56 (15.9)	<0.001*
Hyperlipidemia	0 (0)	52 (14.7)	<0.001*
Smoking	2 (2.3)	70 (19.8)	<0.001*
Drinking	0 (0)	42 (11.9)	0.001*
Clinical features, mean ± SD			
Heart rate, bpm	77.75 ± 9.78	78.48 ± 6.306	0.509
SBP, mmHg	123.48 ± 11.74	132.39 ± 12.73	<0.001*
DBP, mmHg	78.22 ± 8.07	81.79 ± 9.381	0.001*
BMI, kg/m2	23.99 ± 3.39	25.46 ± 4.54	0.005*
Laboratory examinations, median (IQR)			
WBC count, 10^9^/L	6.03 (1.90)	6.81 (2.48)	<0.001*
LY count, 10^9^/L	1.91 (0.72)	1.92 (0.88)	0.208
NEUT count, 10^9^/L	3.43 (1.62)	4.19 (1.90)	<0.001*
MONO count, 10^9^/L	0.35 (0.14)	0.35 (0.17)	0.339
PLT count, 10^9^/L	233.50 (90.50)	248.00 (76.00)	0.309
GLU, mmol/L	5.04 (0.62)	5.10 (1.05)	0.211
ALB, g/L	44.95 (3.18)	45.50 (3.95)	0.364
Cr, μmol/L	57.80 (19.35)	54.80 (21.00)	0.169
UA, μmol/L	310.50 (103.93)	305.60 (113.90)	0.677
TG, mmol/L	0.86 (0.62)	1.20 (0.81)	<0.001*
TC, mmol/L	4.61 (0.95)	4.23 (1.27)	<0.001*
HDL-C, mmol/L	1.53 (0.40)	1.31 (0.36)	<0.001*
LDL-C, mmol/L	2.69 (0.85)	2.40 (1.12)	0.001*
ApoA, g/L	1.39 (0.27)	1.30 (0.30)	<0.001*
ApoB, g/L	0.77 (0.26)	0.82 (0.27)	0.221
Hcy, μmol/L	10.61 (3.98)	12.00 (5.80)	0.001*
NLR	1.78 (0.92)	2.13 (1.17)	0.001*
MLR	0.19 (0.10)	0.19 (0.10)	0.775
PLR	127.13 (77.36)	127.18 (56.82)	0.862
SII	421.84 (291.10)	535.49 (382.41)	0.001*
MHR	0.24 (0.11)	0.27 (0.17)	<0.001*
MetO, μmol/L, median (IQR)	2.40 (0.22)	2.52 (0.35)	<0.001*

HC, healthy control; MMD, moyamoya disease; SD, standard deviation; SBP, systolic blood pressure; DBP, diastolic blood pressure; BMI, body mass index; IQR, interquartile range; WBC, white blood cell; LY, lymphocyte; NEUT, neutrophil; MONO, monocyte; PLT, Platelet; GLU, glucose; ALB, albumin; Cr, creatinine; UA, uric acid; TG, triglyceride; TC, total cholesterol; HDL-C, high-density lipoprotein cholesterol; LDL-C, low-density lipoprotein cholesterol; ApoA, apolipoprotein A; ApoB, apolipoprotein B; Hcy, homocysteine; NLR, neutrophil-to-lymphocyte ratio; MLR, monocyte-to-lymphocyte ratio; PLR, platelet-to-lymphocyte ratio; SII, systemic immune-inflammation index; MHR, monocyte-to-HDL cholesterol ratio; MetO, methionine sulfoxide.

* *p* < 0.05, significant difference.

### Statistical analysis

The SPSS software (version 26.0) and R project (version 3.6.3) were used for all statistical analyzes. Continuous data were compared using t-tests or Mann–Whitney U tests between two groups. Kruskal-Wallis tests or one-way ANOVA were used for comparisons among multiple groups. Continuous data were presented as mean with standard deviation (SD) or median with interquartile range (IQR). Categorical variables were compared with Pearson chi-square tests, Fisher exact tests, and Kruskal-Wallis tests, and categorical variables were presented as frequencies. Spearman correlation tests were used to assess the association between clinical characteristics and MetO quartiles. We have performed three logistic regression models to analyze the role of MetO in MMD and its subtypes, including Crude model, Model 1, and Model 2, which have been described in previous studies (Ge et al., 2022; Zeng et al., 2022). Crude model was the unadjusted regression model of MetO. Model 1 was adjusted for age, gender, heart rate, SBP, DBP, and BMI. Model 2 was further adjusted for WBC count, LY count, NEUT count, MONO count, PLT count, GLU, ALB, Cr, UA, TG, TC, HDL-C, LDL-C, ApoA, ApoB, Hcy, NLR, MLR, PLR, SII, and MHR. Receiver-operating characteristic (ROC) curves and area under curve (AUC) values were used to evaluate the predictive ability of the models for the risk of MMD and its subtypes. The area under curve (AUC) has been calculated. For all analyzes, a two-sided *p* value<0.05 was considered statistically significant.

## Results

### Baseline characteristics of study participants

**Figure 2:**
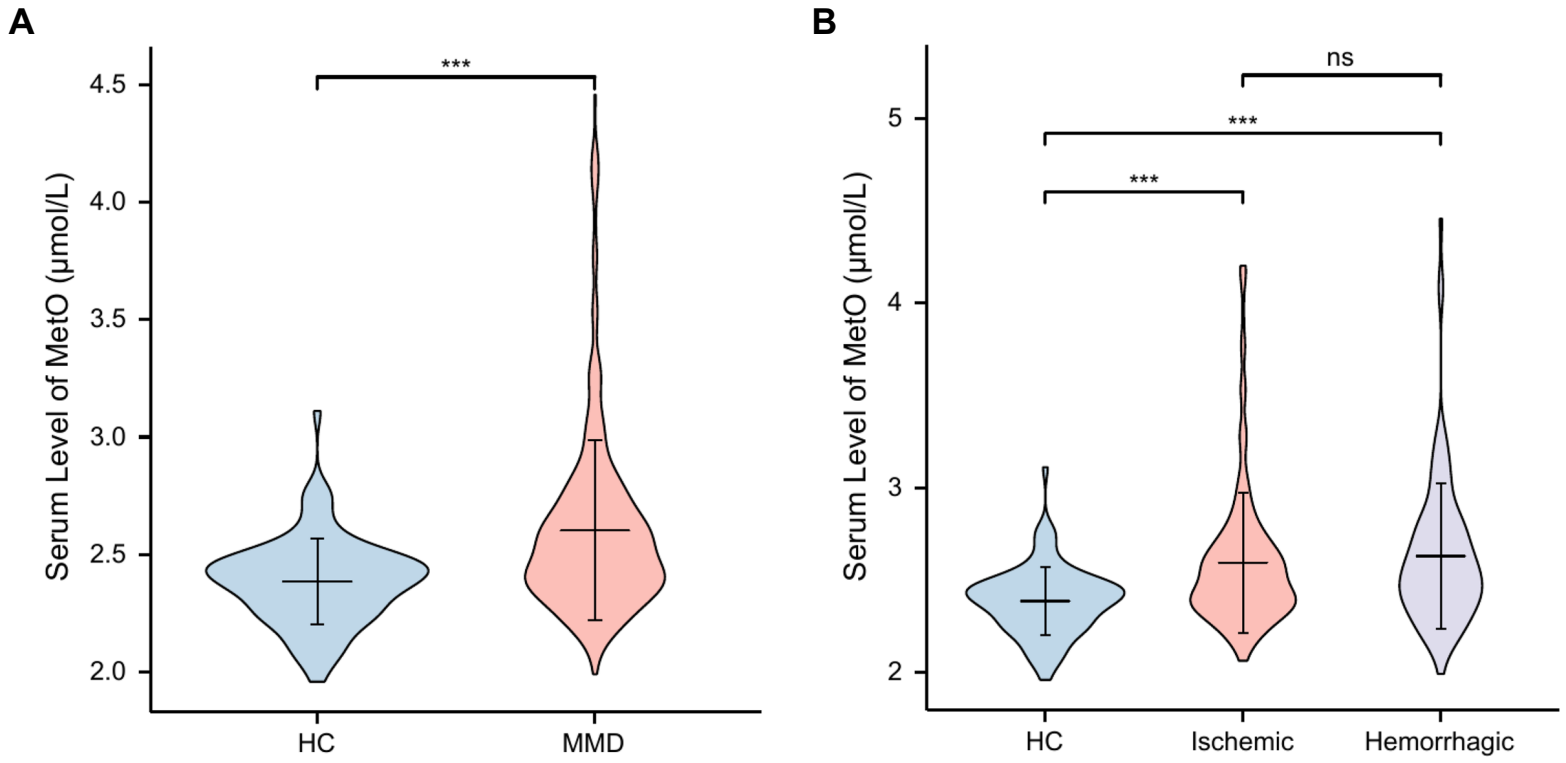
Levels of MetO between HCs and MMD and its subtypes. ns, not significant; ***, *p* < 0.001. **(A)** Comparison of MetO levels between HCs and MMD patients. **(B)** Comparison of MetO levels between HCs and MMD subtypes.

In this study, a total of 353 MMD patients (253 ischemic-type, 100 hemorrhagic-type) and 88 age-matched HCs were eventually included for analysis. Baseline variables were compared between HCs and MMD patients (Table 1). There were no significant differences in age and gender (*p* > 0.05). However, rates of hypertension, diabetes, hyperlipidemia, smoking, and drinking were significantly higher in the MMD group than in the HC group (*p* < 0.05 for all). Regarding clinical features, the MMD group had significantly higher levels of SBP (*p* < 0.001), DBP (*p* = 0.001), and BMI (*p* = 0.005). Additionally, levels of inflammatory biomarkers were significantly higher in the MMD group, including WBC count, NEUT count, NLR, SII, and MHR (p < 0.05 for all). There were also more risk factors in the MMD group, including significantly higher levels of TG, Hcy, and lower levels of HDL-C (*p* < 0.05 for all). In addition, the MMD group had significantly higher levels of MetO compared to the HCs (*p* < 0.001; Figure 2A).

We also compared these variables between HCs and MMD subtypes (Table 2), and the results were similar. Moreover, the level of glucose in the ischemic-type MMD group was significantly higher than in the HC group (*p* = 0.015), indicating a higher risk for ischemia. Levels of MetO were significantly higher in both ischemic-type and hemorrhagic-type MMD groups than in the HC group (*p* < 0.001 for both; Figure 2B).

**Table 2:** Baseline characteristics between HCs and MMD subtypes.

Variables	Health Control (n = 88)	Ischemic MMD (n = 253)	*P* value	Hemorrhagic MMD (n = 100)	P value
Age, y, mean ± SD	39.75 ± 11.62	41.60 ± 10.14	0.186	41.82 ± 10.66	0.204
Gender, Female, *n* (%)	51 (58.0)	139 (54.9)	0.624	66 (66.0)	0.256
Medical History, *n* (%)					
Hypertension	0 (0)	100 (39.5)	<0.001*	29 (29.0)	<0.001*
Diabetes	0 (0)	52 (20.6)	<0.001*	4 (4.0)	0.124
Hyperlipidemia	0 (0)	43 (17.0)	<0.001*	9 (9.0)	0.004*
Smoking	2 (2.3)	53 (20.9)	<0.001*	17 (17.0)	0.001*
Drinking	0 (0)	34 (13.4)	<0.001*	8 (8.0)	0.008*
Clinical features, mean ± SD					
Heart rate, bpm	77.75 ± 9.78	78.23 ± 6.55	0.672	79.11 ± 5.64	0.253
SBP, mmHg	123.48 ± 11.74	133.71 ± 12.69	<0.001*	129.06 ± 12.27	0.002*
DBP, mmHg	78.22 ± 8.07	82.40 ± 9.49	<0.001*	80.23 ± 8.96	0.109
BMI, kg/m2	23.99 ± 3.39	25.91 ± 4.62	<0.001*	24.31 ± 4.13	0.572
Laboratory examinations, median (IQR)					
WBC count, 10^9^/L	6.03 (1.90)	6.96 (2.46)	<0.001*	6.45 (2.49)	0.016*
LY count, 10^9^/L	1.91 (0.72)	2.04 (0.88)	0.017*	1.72 (0.84)	0.139
NEUT count, 10^9^/L	3.43 (1.62)	4.27 (1.84)	<0.001*	3.88 (1.90)	0.003*
MONO count, 10^9^/L	0.35 (0.14)	0.35 (0.17)	0.186	0.35 (0.17)	0.934
PLT count, 10^9^/L	233.50 (90.50)	250.00 (74.50)	0.166	243.50 (74.25)	0.966
GLU, mmol/L	5.04 (0.62)	5.16 (1.13)	0.015*	4.91 (0.67)	0.104
ALB, g/L	44.95 (3.18)	45.50 (4.10)	0.308	45.30 (3.58)	0.671
Cr, μmol/L	57.80 (19.35)	55.70 (21.05)	0.317	53.25 (21.88)	0.070
UA, μmol/L	310.50 (103.93)	312.00 (118.85)	0.304	295.35 (111.83)	0.316
TG, mmol/L	0.86 (0.62)	1.22 (0.79)	<0.001*	1.14 (0.87)	0.008*
TC, mmol/L	4.61 (0.95)	4.11 (1.32)	<0.001*	4.37 (1.08)	0.271
HDL-C, mmol/L	1.53 (0.40)	1.27 (0.39)	<0.001*	1.34 (0.35)	0.001*
LDL-C, mmol/L	2.69 (0.85)	2.25 (1.13)	<0.001*	2.55 (1.08)	0.897
ApoA, g/L	1.39 (0.27)	1.30 (0.32)	<0.001*	1.30 (0.29)	0.008*
ApoB, g/L	0.77 (0.26)	0.82 (0.27)	0.507	0.82 (0.31)	0.036*
Hcy, μmol/L	10.61 (3.98)	12.00 (6.34)	0.001*	11.95 (5.01)	0.005*
NLR	1.78 (0.92)	2.13 (1.17)	0.003*	2.29 (1.40)	0.003*
MLR	0.19 (0.10)	0.19 (0.10)	0.789	0.20 (0.12)	0.163
PLR	127.13 (77.36)	127.18 (56.82)	0.418	144.17 (73.90)	0.218
SII	421.84 (291.10)	535.49 (382.41)	0.002*	541.80 (453.37)	0.004*
MHR	0.24 (0.11)	0.27 (0.173)	<0.001*	0.24 (0.16)	0.122
MetO, μmol/L, median (IQR)	2.40 (0.22)	2.51 (0.33)	<0.001*	2.55 (0.40)	<0.001*

* *p* < 0.05, significant difference.

### Characteristics of MMD patients with different MetO levels

The MMD patients were categorized into two groups based on the median level of MetO, including low-MetO group and high-MetO group. The clinical characteristics of MMD patients in both groups have been shown (Table 3). Although the difference was not statistically significant, patients in the high-MetO group tended to be elder. Furthermore, MMD patients in the high-MetO group had a higher proportion of males, higher levels of BMI, WBC count, NEUT count, MONO count, UA, and MHR. Conversely, these patients had lower levels of HDL-C and ApoA (*p* < 0.05 for all).

**Table 3:** Baseline characteristics of MMD patients between low and high MetO groups.

Variables	Low MetO (1.99–2.47, n = 176)	High MetO (2.47–4.46, n = 177)	P value
Age, y, mean ± SD	40.80 ± 9.97	42.53 ± 10.52	0.114
Gender, Female, *n* (%)	114 (64.8)	91 (51.4)	0.011*
Medical History, *n* (%)			
Hypertension	59 (33.5)	70 (39.5)	0.240
Diabetes	23 (13.1)	33 (18.6)	0.152
Hyperlipidemia	20 (11.4)	32 (18.1)	0.075
Smoking	35 (19.9)	35 (19.8)	0.979
Drinking	20 (11.4)	22 (12.4)	0.757
Clinical features, mean ± SD			
Heart rate, bpm	78.48 ± 6.23	78.47 ± 6.40	0.997
SBP, mmHg	132.14 ± 13.03	132.64 ± 12.45	0.708
DBP, mmHg	82.24 ± 8.92	81.33 ± 9.82	0.362
BMI, kg/m2	24.94 ± 4.09	25.98 ± 4.91	0.030*
RNF213 p.R4810K, *n* (%)			0.669
Wild-type	129 (82.2)	122 (80.3)	
Mutant	28 (17.8)	30 (19.7)	
Laboratory examinations, median (IQR)			
WBC count, 10^9^/L	6.64 (2.22)	6.92 (2.59)	0.015*
LY count, 10^9^/L	1.88 (0.84)	1.99 (0.93)	0.162
NEUT count, 10^9^/L	4.12 (1.87)	4.25 (1.91)	0.033*
MONO count, 10^9^/L	0.34 (0.14)	0.37 (0.19)	0.014*
PLT count, 10^9^/L	246.50 (77.75)	248.00 (74.00)	0.888
GLU, mmol/L	5.05 (1.00)	5.13 (1.08)	0.189
ALB, g/L	45.70 (4.10)	45.30 (3.80)	0.508
Cr, μmol/L	54.50 (19.13)	55.70 (21.70)	0.423
UA, μmol/L	296.20 (109.43)	325.30 (124.60)	0.003*
TG, mmol/L	1.15 (0.80)	1.27 (0.81)	0.118
TC, mmol/L	4.23 (1.33)	4.23 (1.20)	0.876
HDL-C, mmol/L	1.34 (0.42)	1.28 (0.30)	0.032*
LDL-C, mmol/L	2.35 (1.17)	2.40 (1.11)	0.744
ApoA, g/L	1.33 (0.31)	1.26 (0.30)	0.007*
ApoB, g/L	0.79 (0.30)	0.85 (0.27)	0.093
Hcy, μmol/L	11.47 (5.13)	12.20 (6.47)	0.216
NLR	2.06 (1.13)	2.27 (1.29)	0.225
MLR	0.18 (0.09)	0.20 (0.10)	0.073
PLR	130.90 (53.18)	125.22 (60.13)	0.306
SII	528.08 (331.89)	544.18 (435.89)	0.310
MHR	0.25 (0.16)	0.30 (0.18)	0.003*
MetO, μmol/L, median (IQR)	2.37 (0.15)	2.71 (0.31)	<0.001*
Clinical type, *n* (%)			0.362
Ischemic-type	130 (73.9)	123 (69.5)	
Hemorrhagic-type	46 (26.1)	54 (30.5)	
Admission mRS, *n* (%)			0.986
0–2	159 (90.3)	160 (90.4)	
3–5	17 (9.7)	17 (9.6)	
Suzuki stage, *n* (%)			0.801
1–2	52 (29.5)	48 (27.1)	
3–4	86 (48.9)	92 (52.0)	
5–6	38 (21.6)	37 (20.9)	

* *p* < 0.05, significant difference.

We also compared the clinical variables of MMD patients based on the quartile levels of MetO (Table 4). The results showed a correlation between higher levels of MetO and an increased proportion of females (*p* = 0.006). Moreover, patients with higher levels of MetO were associated with higher levels of BMI, UA, MHR, and lower levels of HDL-C and ApoA (*p* < 0.05 for all).

**Table 4:** Baseline characteristics of MMD patients according to quartiles of MetO.

Variables	MetO				*p* value	Spearman coefficient	*p* value
	Q1 (1.99–2.37, *n* = 88)	Q2 (2.37–2.52, *n* = 88)	Q3 (2.52–2.71, *n* = 88)	Q4 (2.71–4.46, *n* = 89)			
Age, y, mean ± SD	39.52 ± 9.65	42.07 ± 10.18	42.35 ± 10.47	42.70 ± 10.63	0.154	0.114	0.032*
Gender, Female, *n* (%)	58 (65.9)	56 (63.6)	49 (55.7)	42 (47.2)	0.049*	0.146	0.006*
Medical History, *n* (%)							
Hypertension	30 (34.1)	29 (33.0)	35 (39.8)	35 (39.3)	0.704	0.052	0.327
Diabetes	8 (9.1)	15 (17.0)	18 (20.5)	15 (16.9)	0.207	0.081	0.127
Hyperlipidemia	11 (12.5)	9 (10.2)	14 (15.9)	18 (20.2)	0.262	0.091	0.087
Smoking	17 (19.3)	18 (20.5)	14 (15.9)	21 (23.6)	0.642	0.024	0.659
Drinking	9 (10.2)	11 (12.5)	9 (10.2)	13 (14.6)	0.773	0.038	0.480
Clinical features, mean ± SD							
Heart rate, bpm	78.73 ± 5.59	78.23 ± 6.83	77.69 ± 5.53	79.25 ± 7.10	0.399	0.041	0.444
SBP, mmHg	134.15 ± 13.14	130.13 ± 12.69	130.28 ± 11.70	134.98 ± 12.79	0.014*	0.060	0.262
DBP, mmHg	82.72 ± 9.54	81.77 ± 8.28	80.68 ± 10.14	81.98 ± 9.51	0.549	−0.027	0.619
BMI, kg/m2	24.68 ± 4.40	25.19 ± 3.76	25.41 ± 4.29	26.54 ± 5.42	0.046*	0.121	0.023*
RNF213 p.R4810K, *n* (%)					0.951	0.026	0.652
Wild-type	65 (83.3)	64 (81.0)	59 (79.7)	63 (80.8)			
Mutant	13 (16.7)	15 (19.0)	15 (20.3)	15 (19.2)			
Laboratory examinations, median (IQR)							
WBC count, 10^9^/L	6.75 (2.20)	6.50 (2.32)	6.78 (2.32)	7.05 (2.32)	0.102	0.127	0.017*
LY count, 10^9^/L	1.79 (0.89)	1.97 (0.83)	2.09 (0.83)	1.91 (0.83)	0.294	0.071	0.183
NEUT count, 10^9^/L	4.14 (1.85)	4.06 (1.87)	4.11 (1.87)	4.36 (1.87)	0.143	0.107	0.045*
MONO count, 10^9^/L	0.33 (0.14)	0.34 (0.16)	0.37 (0.16)	0.37 (0.16)	0.068	0.142	0.008*
PLT count, 10^9^/L	245.50 (73.50)	247.50 (79.50)	249.00 (79.50)	248.00 (79.50)	0.895	<0.001	0.995
GLU, mmol/L	5.05 (0.96)	5.08 (1.06)	5.16 (1.06)	5.12 (1.06)	0.608	0.067	0.208
ALB, g/L	46.45 (3.40)	44.75 (3.60)	45.45 (3.60)	45.30 (3.60)	0.015*	−0.082	0.126
Cr, μmol/L	54.60 (20.88)	54.40 (17.85)	53.65 (17.85)	59.60 (17.85)	0.281	0.070	0.192
UA, μmol/L	293.05 (115.93)	298.65 (98.45)	305.10 (98.45)	347.50 (98.45)	0.004*	0.180	0.001*
TG, mmol/L	1.15 (0.85)	1.17 (0.88)	1.32 (0.88)	1.25 (0.88)	0.239	0.072	0.180
TC, mmol/L	4.36 (1.40)	4.18 (1.37)	4.29 (1.37)	4.20 (1.37)	0.703	−0.033	0.532
HDL-C, mmol/L	1.44 (0.48)	1.28 (0.38)	1.29 (0.38)	1.28 (0.38)	0.011*	−0.154	0.004*
LDL-C, mmol/L	2.36 (1.25)	2.32 (1.12)	2.48 (1.12)	2.37 (1.12)	0.741	−0.004	0.938
ApoA, g/L	1.37 (0.38)	1.30 (0.27)	1.28 (0.27)	1.24 (0.27)	0.001*	−0.201	<0.001*
ApoB, g/L	0.78 (0.28)	0.82 (0.29)	0.87 (0.29)	0.82 (0.29)	0.231	0.085	0.112
Hcy, μmol/L	11.79 (5.53)	11.38 (4.77)	11.95 (4.77)	12.60 (4.77)	0.592	0.067	0.211
NLR	2.11 (1.14)	2.03 (0.77)	2.24 (0.77)	2.31 (0.77)	0.407	0.056	0.298
MLR	0.18 (0.09)	0.18 (0.10)	0.19 (0.10)	0.20 (0.10)	0.264	0.099	0.062
PLR	135.42 (53.20)	125.53 (52.08)	120.93 (52.08)	125.97 (52.08)	0.624	−0.048	0.365
SII	600.87 (338.74)	507.29 (286.33)	528.39 (286.33)	544.91 (286.33)	0.620	0.043	0.426
MHR	0.24 (0.15)	0.27 (0.16)	0.30 (0.16)	0.31 (0.16)	0.004*	0.188	<0.001*
MetO, μmol/L, median (IQR)	2.28 (0.12)	2.43 (0.07)	2.60 (0.07)	2.89 (0.07)	<0.001*	0.968	<0.001*
Clinical type, n (%)					0.167	0.088	0.100
Ischemic-type	67 (76.1)	63 (71.6)	67 (76.1)	56 (62.9)			
Hemorrhagic-type	21 (23.9)	25 (28.4)	21 (23.9)	33 (37.1)			
Admission mRS, n (%)					0.356	0.033	0.536
0–2	83 (94.3)	76 (86.4)	80 (90.9)	80 (89.9)			
3–5	5 (5.7)	12 (13.6)	8 (9.1)	9 (10.1)			
Suzuki stage, n (%)					0.335	−0.017	0.754
1–2	22 (25.0)	30 (34.1)	19 (21.6)	29 (32.6)			
3–4	52 (59.1)	34 (38.6)	47 (53.4)	45 (50.6)			
5–6	14 (15.9)	24 (27.3)	22 (25.0)	15 (16.9)			

* *p* < 0.05, significant difference.

### Association between MetO and MMD

**Figure 3:**
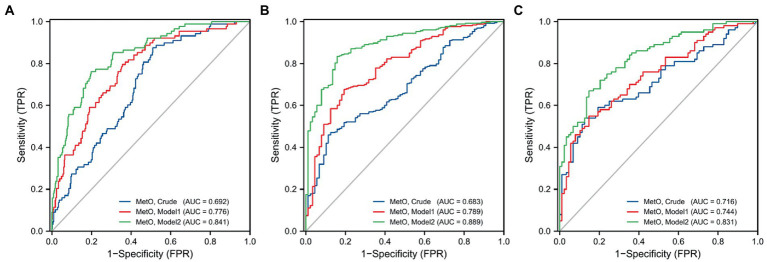
ROC curves of MetO in different models for the risk of MMD and its subtypes. **(A)** MMD overall; **(B)** Ischemic-type; **(C)** Hemorrhagic-type.

In this study, we observed a positive association between serum MetO and the risk of MMD in Crude model (OR: 28.695, 95%CI: 8.033–102.500, *p* < 0.001; Table 5). In Model 1, the risk of MMD increased with each increment in MetO level (OR: 30.332, 95%CI: 7.812–117.770, *p* < 0.001). In Model 2, MetO was also found to increase the risk of MMD (OR: 20.215, 95%CI: 4.842–84.397, *p* < 0.001). The ROC curves revealed that the predictive accuracy of Model 2 (AUC = 0.841; Figure 3A) significantly improved compared to Crude model (AUC = 0.692) and Model 1 (AUC = 0.776). Similar results were found in ROC curves of three models for MMD subtypes (Table 5; Figures 3B,C).

**Table 5:** Association between different MetO levels and the risk of MMD and its subtypes.

MetO	No. of events (%)	Crude model		Model 1		Model 2	
		OR (95%CI)	*p* value	OR (95%CI)	*p* value	OR (95%CI)	*p* value
MMD overall							
Continuous	353 (80.0)	28.695 (8.033–102.500)	<0.001*	30.332 (7.812–117.770)	<0.001*	20.215 (4.842–84.397)	<0.001*
MetO level							
Low (1.96–2.47)	155 (70.1)	Ref		Ref		Ref	
High (2.47–4.46)	198 (90.0)	3.832 (2.264–6.487)	<0.001*	3.699 (2.142–6.389)	<0.001*	2.888 (1.613–5.170)	<0.001*
Ischemic MMD							
Continuous	253 (74.2)	27.741 (7.093–108.485)	<0.001*	27.900 (6.231–124.915)	<0.001*	19.993 (3.560–112.281)	0.001*
MetO level							
Low (1.96–2.47)	117 (63.9)	Ref		Ref		Ref	
High (2.47–4.46)	136 (86.1)	3.487 (2.028–5.997)	<0.001*	3.153 (1.766–5.631)	<0.001*	2.474 (1.290–4.745)	0.006*
Hemorrhagic MMD							
Continuous	100 (53.2)	38.917 (8.462–178.970)	<0.001*	33.564 (7.208–156.300)	<0.001*	49.665 (8.380–294.345)	<0.001*
MetO level							
Low (1.96–2.47)	38 (36.5)	Ref		Ref		Ref	
High (2.47–4.46)	62 (73.8)	4.895 (2.609–9.183)	<0.001*	4.746 (2.494–9.033)	<0.001*	4.340 (2.147–8.772)	<0.001*

* *p* < 0.05, significant difference.

**Figure 4:**
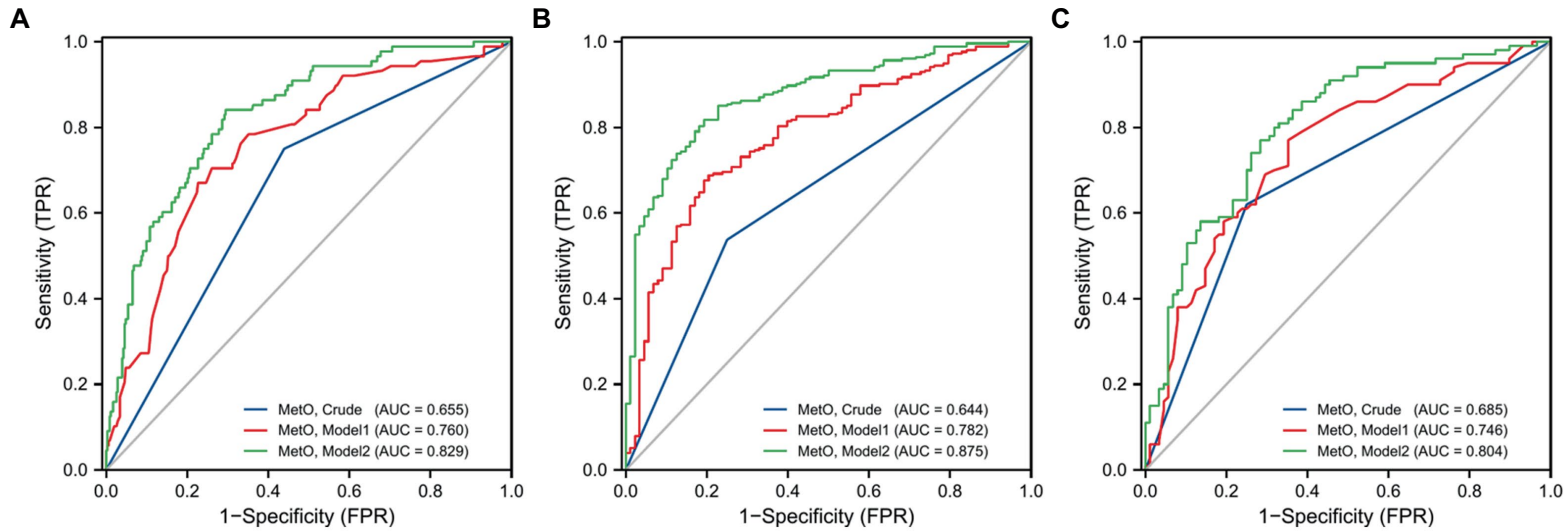
ROC curves of low and high levels of MetO in different models for the risk of MMD and its subtypes. **(A)** MMD overall; **(B)** Ischemic-type; **(C)** Hemorrhagic-type.

We also compared the risk of MMD between low and high MetO levels. We found cases with high MetO level had a significantly higher risk of MMD in Crude model (OR: 3.832, 95%CI: 2.264–6.487, *p* < 0.001), Model 1 (OR: 3.699, 95%CI: 2.142–6.389, *p* < 0.001), and Model 2 (OR: 2.888, 95%CI: 1.613–5.170, *p* < 0.001; Table 5). The AUC values showed an improvement in Crude model (AUC = 0.655; Figure 4A), Model 1 (AUC = 0.760), and Model 2 (AUC = 0.829). Similar results were observed in Crude model, Model 1, and Model 2 of ischemic and hemorrhagic MMD (Figures 4B,C).

**Figure 5:**
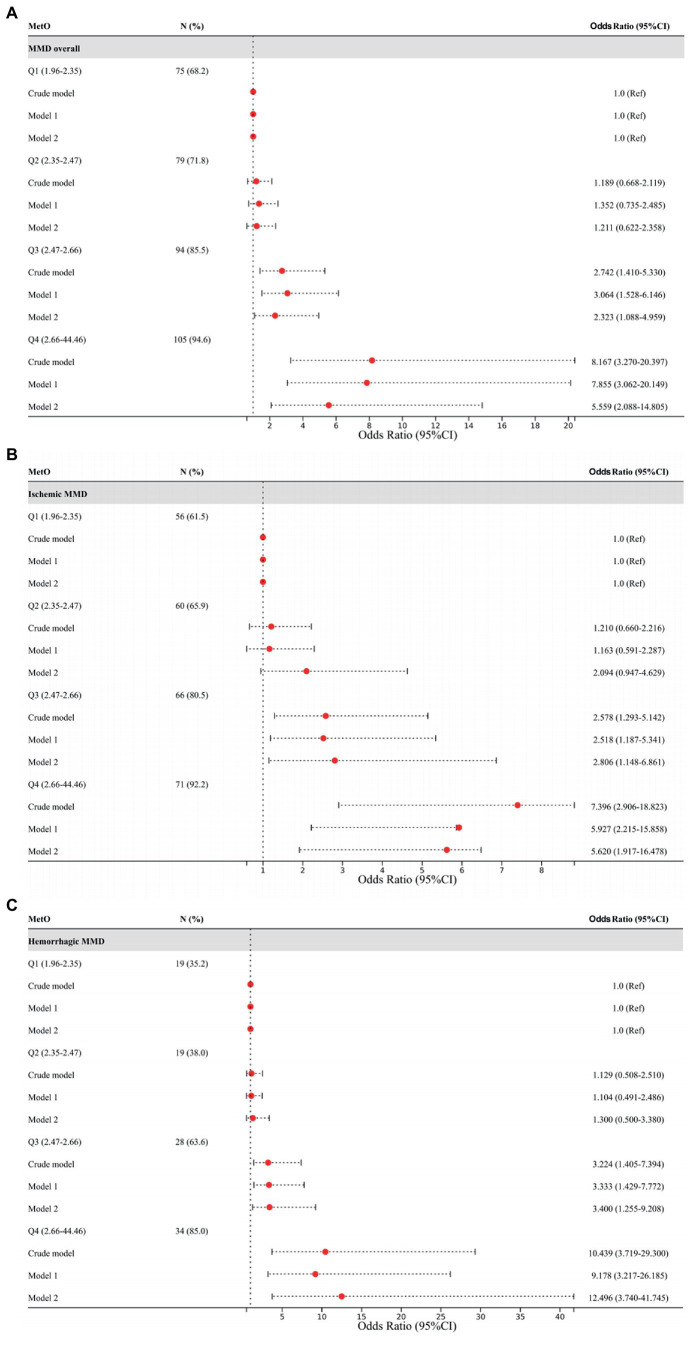
Association between different quartiles of MetO and the risk of MMD and its subtypes. **(A)** MMD overall; **(B)** Ischemic-type; **(C)** Hemorrhagic-type.

**Figure 6:**
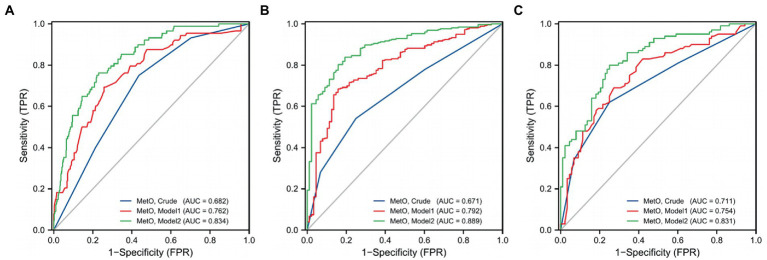
ROC curves of MetO quartiles in different models for the risk of MMD and its subtypes. **(A)** MMD overall; **(B)** Ischemic-type; **(C)** Hemorrhagic-type.

Furthermore, we assessed MetO as quartiles and analyzed its risk in MMD. We found that the proportion of MMD events increased with the rise of MetO quartiles (Figures 5A–C). In contrast to Q1 of MetO, cases in Q3 and Q4 of MetO had a significantly higher risk of MMD in Crude model (Q3, OR: 2.742, 95%CI: 1.410–5.330, *p* = 0.003; Q4, OR: 8.167, 95%CI: 3.270–20.397, *p* < 0.001), Model 1 (Q3, OR: 3.064, 95%CI: 1.528–6.146, *p* = 0.002; Q4, OR: 7.855, 95%CI: 3.062–20.149, *p* < 0.001), and Model 2 (Q3, OR: 2.323, 95%CI: 1.088–4.959, *p* = 0.029; Q4, OR: 5.559, 95%CI: 2.088–14.805, *p* = 0.001). The AUC value of ROC curves increased with the development of the models (Crude model, AUC = 0.682; Model 1, AUC = 0.762; Model2, AUC = 0.834 Figure 6A). Consistent with MMD overall, the risk of ischemic and hemorrhagic MMD increased with increasing MetO quartiles. In comparison to the lowest quartile (Q1), cases in the third (Q3) and fourth (Q4) MetO quartiles showed a significantly correlation with ischemic MMD in Crude model (Q3, OR: 2.578, 95%CI: 1.293–5.142, *p* = 0.042; Q4, OR: 7.396, 95%CI: 2.906–18.823, *p* < 0.001), Model 1 (Q3, OR: 2.518, 95%CI: 1.187–5.341, *p* = 0.016; Q4, OR: 5.927, 95%CI: 2.215–15.858, *p* < 0.001), and Model 2 (Q3, OR: 2.806, 95%CI: 1.148–6.861, *p* = 0.024; Q4, OR: 5.620, 95%CI: 1.917–16.478, *p* = 0.002). The AUC of ROC curves in Crude model, Model 1, and Model 2 were 0.671, 0.792, and 0.889, respectively (Figure 6B). Similarly, the third (Q3) and fourth (Q4) MetO quartiles were significantly correlated with the risk of hemorrhagic MMD in Crude model (Q3, OR: 3.224, 95%CI: 1.405–7.394, *p* = 0.006; Q4, OR: 10.439, 95%CI: 3.719–29.300, p < 0.001), Model 1 (Q3, OR: 3.333, 95%CI: 1.429–7.772, *p* = 0.005; Q4, OR: 9.178, 95%CI: 3.217–26.185, *p* < 0.001), and Model 2 (Q3, OR: 3.400, 95%CI: 1.255–9.208, *p* = 0.016; Q4, OR: 12.496, 95%CI: 3.740–41.745, *p* < 0.001). The AUC of ROC curves in Crude model, Model 1, and Model 2 were 0.711, 0.754, and 0.831, respectively (Figure 6C).

## Discussion

In this prospective study, we compared the difference in clinical characteristics between MMD patients and HCs. We also investigated the differences between MMD subtypes and HCs. Our findings revealed that serum MetO levels were significantly higher in the MMD group and its subtypes than those in the HC group. Subsequently, we analyzed the relationship between MetO and the risk of MMD and found that MetO was an independent risk factor. The risk of MMD increased with the elevation of MetO levels. This study indicated that MetO might play a crucial role in the pathogenesis and progression of MMD.

Met, as an essential amino acid, was highly susceptible to oxidation. Under physiological and pathological oxidative stress conditions, reactive oxygen species (ROS) result in the oxidation of Met to MetO, including two stereoisomers, S-MetO and R-MetO (Brot et al., 1981; Moskovitz et al., 1996; Makukhin et al., 2016). The increase in MetO levels result in the accumulation of toxic proteins and alterations in cellular functions (Moskovitz and Smith, 2021). MetO is an attractive biomarker for oxidative stress and an important indicator for its assessment (Gu et al., 2015). The MetO reductase system could specifically reverse MetO to Met (Moskovitz et al., 2000; Grimaud et al., 2001; Lourenço Dos Santos et al., 2018). The MetO reductase system played an important role in regulating protein functions, signaling pathways, and repairing oxidative damage proteins. Several studies have confirmed that the enhanced function of the MetO reductase system could increase cellular resistance to oxidative stress and related diseases (Moskovitz et al., 1998, 2001; Lee et al., 2005). Elevated levels of MetO were associated with neurodegenerative diseases, cancers, cardiovascular disease, stroke, and other diseases (Gu et al., 2015; Reiterer et al., 2019). However, the metabolomics of patients with MMD has not been extensively studied. In this study, we found that serum MetO levels in MMD patients were significantly higher than those in HCs, and MetO was positively associated with the risk of MMD.

The link between MetO and cerebrovascular disease has been shown. A recent large prospective study on metabolomic characteristics of ischemic stroke found a significant association between MetO and incident stroke, with higher levels being associated with increased risk (Balasubramanian et al., 2022). Another study demonstrated that betaine could decrease proinflammatory cytokine production and reduce oxidative stress after brain ischemia and reperfusion injury by upragulating MetO reductase (Li et al., 2022). Moreover, the formation of MetO in von Willebrand factor was found to inhibit the ADAMTS-13 cleavage, promoting thrombosis in oxidative stress-related diseases (Lancellotti et al., 2010).

MMD was considered to result from a combination of inflammation, genetic, and other factors. Previous evidence showed that levels of oxidative stress in endothelial colony-forming cells were significantly increased in MMD patients (Choi et al., 2018). By eliminating ROS, the endothelial cells showed an improvement in angiogenesis capacity. It indicated that MMD was an oxidative stress-related disease with chronic inflammatory response. A previous study found that overexpression of MetO reductase A could reduce MetO in calcium/calmodulin-dependent protein kinase II, attenuating ROS-augmented NF-κB activation in endothelial cells (Gu et al., 2016). Another study showed that MetO reductase played an antioxidant role in vascular smooth muscle cells and prevented oxidative damage in cytoplasm and mitochondria (Haenold et al., 2008). In addition, consistent with the increase of MetO levels, vasodilatative activity decreased and vasoconstrictive mediators significantly increased (Pichler Hefti et al., 2013). Although the precise mechanism by which MetO contributed to MMD remained unclear, it was hypothesized that high levels of MetO induced the dysfunction in vessel-associated cells and the dysregulation in vasoactive substances, which might be involved in MMD pathogenesis.

Oxidative stress was generally accompanied by inflammation responses characterized by the production of ROS and the release of inflammatory cytokines. MetO has been shown to induce the activation of M1/classical macrophage activation, alter the extracellular nucleotide metabolism, and promote the increase of macrophage ATPase/ADPase activity (Dos Santos et al., 2017). The MetO reductase system might play a positive role in antioxidant defense and inflammation-related damage signal transduction pathways. Overexpression of MetO reductase A in microglia cells led to the reduction in lipopolysaccharide (LPS)-induced activation of ROS/MAPKs/NF-κB signaling pathways (Fan et al., 2015). Silencing MetO reductase A led to the activation of microglia activation and the production of Iba1, TNF-α, IL-1β, ROS, and NOX2 (Fan et al., 2020). Additionally, MetO reductase B1 activated the transcription-6 (STAT6) pathway after immunization and promoted the differentiation of T-helper cells type 1 and follicular helper T cells (Lee et al., 2017). Furthermore, MetO reductase deficiency could cause vascular smooth muscle cell proliferation and neointimal hyperplasia after vascular injury (Klutho et al., 2015). Therefore, we speculated that the combination of immune response disorder and abnormal intimal hyperplasia caused by MetO reductase deficiency might be the underlying cause of MMD.

Our previous study has shown that several factors, such as ALB, BMI and HDL-C, were associated with the risk of MMD (Ge et al., 2020). In this study, we found that some of these factors had the same trend with MetO levels, including BMI, uric acid, HDL-C, and ApoA. These results indicated that MetO was associated with the dysregulation of lipid metabolism in MMD. Moreover, our study revealed that inflammatory factors were mostly elevated in high MetO groups, which was consistent with the increasing levels of inflammatory biomarkers in MMD and its subtypes compared to HCs. In conclusion, MetO might have an impact on vascular damage through dyslipidemia and chronic inflammatory responses. After adjusting for potential confounders in multivariate regression models, we found that MetO was still significantly associated with the risk of MMD overall, ischemic-types, and hemorrhagic-types. These results highlighted the importance of MetO downregulation, and suggested that MetO reductases might serve as potential therapeutic targets for MMD. However, there were still several limitations in our study. Firstly, Due to the nature of single-center, the number of participants included in our study was relatively limited. Large multi-center prospective studies were needed for further validation. Secondly, the participants in our study were all adults. It was still unclear whether the findings were consistent in pediatric patients. Thirdly, the dietary intake information of participants was not collected in the study, which might affect the outcome. Fourthly, the regulatory mechanism and signaling pathways of MetO in MMD needed further studies *in vitro* and *in vivo*.

## Conclusion

In summary, our study revealed that the levels of MetO was increased in MMD patients, and we identified MetO as an independent risk factor for MMD and its subtypes. These findings suggested that MetO reductases might serve as potential therapeutic targets. Our study provided a novel perspective on the risk of MMD.

## Data availability statement

The raw data supporting the conclusions of this article will be made available by the authors, without undue reservation.

## Ethics statement

The studies involving human participants were reviewed and approved by Ethics Committee of Beijing Tiantan Hospital, Capital Medical University. The patients/participants provided their written informed consent to participate in this study.

## Author contributions

JL illustrated all the results and drafted the manuscript. PG and CZ performed all the statistical analyzes. QH, CL, CT, and YuZ collected the data. JW, QZ, RW, and YaZ revised the manuscript. DZ and JZ conceived and designed this research. All authors have read and approved the final version of the manuscript.

## Funding

This study was supported by the National Natural Science Foundation of China (81701137), the National Key Research and Development Program of China (2021YFC2500502), Beijing Municipal Organization Department Talents Project (2015000021469G219), and Beijing Municipal Administration of Hospitals’ Mission Plan (SML20150501).

## Conflict of interest

The authors declare that the research was conducted in the absence of any commercial or financial relationships that could be construed as a potential conflict of interest.

## Publisher’s note

All claims expressed in this article are solely those of the authors and do not necessarily represent those of their affiliated organizations, or those of the publisher, the editors and the reviewers. Any product that may be evaluated in this article, or claim that may be made by its manufacturer, is not guaranteed or endorsed by the publisher.
